# Closed-Loop Targeted Memory Reactivation during Sleep Improves Spatial Navigation

**DOI:** 10.3389/fnhum.2018.00028

**Published:** 2018-02-06

**Authors:** Renee E. Shimizu, Patrick M. Connolly, Nicola Cellini, Diana M. Armstrong, Lexus T. Hernandez, Rolando Estrada, Mario Aguilar, Michael P. Weisend, Sara C. Mednick, Stephen B. Simons

**Affiliations:** ^1^Teledyne Scientific & Imaging, Durham, NC, United States; ^2^Department of Psychology, University of California, Riverside, Riverside, CA, United States; ^3^Department of General Psychology, University of Padova, Padova, Italy; ^4^Rio Grande Neurosciences, Dayton, OH, United States

**Keywords:** sleep, targeted memory reactivation, closed-loop, spatial memory, navigation, spindles, slow oscillation, slow wave sleep

## Abstract

Sounds associated with newly learned information that are replayed during non-rapid eye movement (NREM) sleep can improve recall in simple tasks. The mechanism for this improvement is presumed to be reactivation of the newly learned memory during sleep when consolidation takes place. We have developed an EEG-based closed-loop system to precisely deliver sensory stimulation at the time of down-state to up-state transitions during NREM sleep. Here, we demonstrate that applying this technology to participants performing a realistic navigation task in virtual reality results in a significant improvement in navigation efficiency after sleep that is accompanied by increases in the spectral power especially in the fast (12–15 Hz) sleep spindle band. Our results show promise for the application of sleep-based interventions to drive improvement in real-world tasks.

## Introduction

Sleep may facilitate the transformation of recent fragile memories into stable long-term memories. Compared with an equivalent period of wake, performance in several memory domains demonstrates a greater magnitude of improvement after sleep (Rasch and Born, 2013). Several electrophysiological features of non-rapid eye movement (NREM) sleep have been linked with memory consolidation, with the majority of these studies focusing on the role of slow wave activity, which refers to the low-frequency oscillations (0.05–4 Hz) that characterize deeper NREM sleep (e.g., Gais et al., 2002; Wilhelm et al., 2014). Slow oscillations (SOs) originate in the cortex and reflect synchronized neural fluctuations between hyperpolarized down-states and depolarized up-states. Spindles, another prominent NREM sleep feature consisting of 9–15 Hz oscillatory bursts, have gained attention for their role in hippocampal-cortical communication and declarative memory consolidation during sleep. Correlational studies have shown that the number of sleep spindles increases following hippocampal-dependent learning (Eschenko et al., 2006) and spindles are temporally coupled with hippocampal sharp wave ripples in rodents (Siapas and Wilson, 1998) and in humans (Staresina et al., 2015). They may facilitate the integration of newly learned information with existing knowledge (Tamminen et al., 2013) and are correlated with better retention of declarative memories in humans (Gais et al., 2002; Schabus et al., 2004; Clemens et al., 2005; Schmidt et al., 2006). A third electrophysiological feature of NREM sleep are hippocampal sharp wave-ripples, short high-frequency bursts that coincide with reactivations of neurons that were active during learning (e.g., Wilson and McNaughton, 1994).

Temporal coupling of SOs, spindles, and hippocampal sharp wave-ripples may be a key mechanism underlying the hippocampal-neocortical dialog characteristic of systems consolidation. The drug zolpidem (Ambien) increased the temporal consistency of spindle occurrences during the down-to-up phase of slow oscillations (Niknazar et al., 2015). Furthermore, later performance improvement was correlated with this spindle/SO timing. This suggests that declarative memory consolidation is facilitated when thalamic spindles coincide with the down-to-up phase of cortical SOs. Thus SOs may provide a top-down temporal frame for these oscillatory events (Crunelli and Hughes, 2010; Lemieux et al., 2014). Specifically, individual hippocampal sharp wave ripple events appear to be nested in the trough of succeeding spindles (Timofeev and Bazhenov, 2005; Staresina et al., 2015), and these spindle-ripple events may represent a bottom-up mechanism whereby reactivated hippocampal memory information (coded in ripples) is passed to spindles, which then reach neocortical networks via the SO (Born et al., 2006; Diekelmann and Born, 2010). Recently, Yordanova et al. (2017) have shown that the temporal coupling of SO up-states and spindles is greater in the hemisphere that had been activated during prior learning.

Targeted memory reactivation (TMR) has been successful in enhancing memory during sleep using external stimulation. The TMR approach associates sensory stimuli (e.g., odor or sound cue) with target information during encoding and then presents the same cues during sleep to facilitate memory consolidation, including visuospatial (Rasch et al., 2007; Rudoy et al., 2009; van Dongen et al., 2012; Creery et al., 2015), verbal memories (Schreiner and Rasch, 2014; Batterink and Paller, 2017) and fear extinction (He et al., 2015). The strength of specific memory enhancement appears to depend on the timing relative to the phase of the SO, although most studies have thus far delivered cues using an open-loop approach during NREM stage 3 sleep (Batterink et al., 2016). Batterink et al. (2016) demonstrated that the largest memory benefit occurred when TMR cues were delivered during the descending phase of the SO down state, which presumably allowed for the cue information to be processed by the cortex and hippocampus during their up states. This suggests that the largest memory benefits may be realized when the cues are delivered during the transition from cortical down states to up states.

In the current study, we developed a novel method to enhance sleep spindles and spatial navigation skills using closed-loop targeted memory reactivation (CL-TMR) time-locked to the down-state to up-state transitions (DUPTs) of SOs. We tested navigation ability at multiple time points across 3 days in order to determine when the benefits of CL-TMR emerge, and how long they are observed. DUPTs were targeted to increase the likelihood of affecting spindles during the rising phase and peak of the SO. In this study, we did not investigate whether the closed-loop cue delivery confers significant benefit when compared with an open-loop approach. Multiple studies suggest that TMR may be most effective when time-locked to the DUPTs (Niknazar et al., 2015; Batterink et al., 2016), and a recent non-peer reviewed study has shown that when cues are delivered during the down state, memory enhancement is superior when compared with the effect observed when cues are delivered during the up state (Göldi et al., unpublished). The goal of this study was to develop a robust methodology for reliably delivering stimuli during DUPTs and to demonstrate that CL-TMR can be used to drive performance gains in ecologically valid, complex learning tasks over longer periods of time.

## Materials and Methods

### Participants

This study was approved by the New England Independent Review Board and all participants gave written informed consent in accordance with the Declaration of Helsinki. Healthy individuals (*N* = 37, 16 women, *M*_age_ = 25.14 years, *SD*_age_ = 5.75 years) were recruited from the surrounding communities in Durham, NC; Dayton, OH, United States; and Riverside, CA, United States. They were instructed to avoid caffeine, alcohol, and naps within 24 h of any study session. Participants arrived at the laboratory around 12 pm. Data from an additional 18 participants (8 women, *M*_age_ = 28.44 years, *SD*_age_ = 7.33 years) were excluded because of one or a combination of the following criteria: they could not complete the VR task inside the headset due to nausea, they did not receive any cues during both naps or only received cues during one of the naps, the data recordings were of poor quality (e.g., extremely noisy record), and/or they did not sleep at least 30 min with at least 15 min in stages 2 or 3. Demographic data of included participants are shown in **Table 1**.

**Table 1:** Demographics of participants included in the analysis.

	Control (*N* = 20)	CL-TMR (*N* = 17)
Mean age (*SD*)	26.25 years (6.56)	23.82 years (4.02)
Number of women	10	6
Ethnicity		
White/Caucasian	15	9
Black/African American	1	–
Hispanic/Latino	3	2
Asian	1	3
Native American	–	1
Biracial or multiracial	–	2
Education		
Less than high school	–	–
High school	3	1
Some college	6	7
Associate degree	–	–
Bachelor degree	5	7
Graduate degree	6	2


### Experimental Design and Statistical Analysis

#### Electroencephalography (EEG) Recording

Electroencephalographic (EEG) data were collected on a Brain Products 32-channel actiCAP electrode system and BrainAmp DC amplifier (Brain Products, GmbH, Munich, Germany) using the standard 10–20 electrode layout. Electrocardiogram (ECG) and electrooculogram (EOG) electrodes were used for offline artifact rejection and assessment of rapid-eye movement (REM) sleep. For EOG, one electrode was placed 1 cm above the corner of the right eye and the second electrode was placed 1 cm below the corner of the left eye following recommended criteria for sleep recording (Iber et al., 2007). The left shoulder blade was used as a common reference. EOG was collected to facilitate offline sleep scoring used in analysis of NREM sleep biomarkers and for reference against automated sleep scoring in the closed-loop system. Active reference electrodes were attached to the left and right mastoid sites with an adhesive ring. All electrophysiological data was collected at a sample rate of 500 Hz and recorded using the BrainVision Recorder software for offline analysis. The frequency boundaries during recording were 0 to 1000 Hz.

#### Navigation Task

**FIGURE 1:**
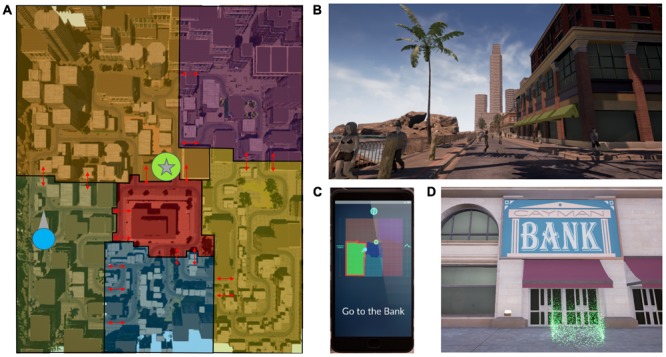
**(A)** The VR environment was divided into six districts, each with its own thematic buildings. Auditory cues were delivered at borders between districts (red arrows) and are border- and heading-specific. **(B)** Two boundary elements are shown in this snapshot from a participant’s point of view, the beach and the skyscraper. **(C)** The cell phone interface provided an aerial schematic of the six districts and their orientation in relation to the beach and the mountains (boundary elements). The participant’s location and heading (blue circle with an arrow) and the target destination location (green circle with a star) were shown. At the beginning of a route, a message directed participants to a target location (e.g., “Go to the Bank”). The cell phone provided feedback if the participant was navigating along a suboptimal route, and oriented the participant to their location as the auditory cues were experienced and boundaries were crossed (see the highlighted border in red as the participant crosses from the dark blue to the green district). **(D)** Upon reaching the target destination, participants stepped into a green portal. The participant would then respawn at the next starting point and the task would begin anew. On **(A)**, the target location from **(D)** (bank) and the location and heading of the participant in B are marked as on the cellphone map with a star enclosed in a green circle, and a blue circle with an arrow, respectively.

The virtual reality (VR) navigation task performed by participants was designed by the authors and implemented into custom-built software by Intific, Inc. (Austin, TX, United States) using the Unreal Engine. Participants wore an Oculus DK2 headset (Oculus VR, LLC, Menlo Park, CA, United States). The VR environment consisted of a large city with six districts; **Figure 1A** shows an overhead map with district outlines. These six districts contained buildings with unique architectural features such that they could potentially help one identify a change in districts when moving from one to another (e.g., there were apartment complexes located in a residential district). Pedestrians were also seen in each district and some wore clothing that could be uniquely attributed to a certain district (e.g., men and women could be seen wearing suits in the financial district).

Boundary landmarks were placed in the environment to encourage the use of a hippocampal-dependent boundary navigation strategy (Doeller et al., 2008; **Figure 1B**). A large skyscraper was placed at the northwest corner and a factory with smokestacks at the southeast corner. The west edge of the map was bordered by a beach and the east edge was bordered by mountains. Within the environment, locations of note had a sign in front with their name or logo, and were places that the participant was required to navigate to or from during the training portion of the task.

A virtual cell phone interface presented a sparsely detailed map such that participants could see their own location and heading, the location of the target destination, and the borders between the six districts (**Figure 1C**). No roads or buildings were shown on the map to prevent explicit route planning. The boundary landmarks to the east and west of the map (mountains and the beach, respectively) were designated on the map, and the cardinal direction north was designated by an “N.” The district containing the navigator’s current location had a highlighted border. The participant’s location was designated by a blue circle on the map with an arrow pointing in the direction of the participant’s heading. The location of the destination landmark was designated by a star encircled in green (**Figures 1A,C**).

At the beginning of a route, the cell phone would present a message directing the participants to a target location (e.g., “Go to the Bank”; **Figure 1C**) and show the target destination’s location on the map. The participant could not call up the cell phone at will. Instead, the phone was automatically called up at the beginning of a route and whenever the participant crossed a boundary between districts. Other than the initial guidance provided by the phone, participants were allowed to freely explore the environment while searching for target locations during the training portions of the task. Additionally, during the training portion of the task, the cell phone was called up to alert the participant if they traveled too far along a non-optimal route. These non-optimal route alerts were turned off during testing intervals of the task. Once participants reached the target destination, they would step into a green portal and respawn at the next route’s starting location (**Figure 1D**). If a participant could not complete the route within a specified time limit, the participant would respawn at the next route’s starting location at the end of the time limit.

Auditory cues that were designed to be contextually appropriate for an urban environment and were delivered as participants crossed district borders (e.g., the sound of a pipe dropping onto the ground was heard in an industrial area). The cues were border and heading-specific. As mentioned previously, the cell phone would be called up and border of the district one was traveling into would be highlighted. This would occur at the same time the auditory cue played. A total of 18 unique cues were experienced by the participants in the environment, and the optimal routes were designed to ensure that each auditory cue would be experienced at least once during training. The cues delivered during the nap were shortened versions (700 ms long) of the cues heard during the navigation task and were naturalistic sounds (e.g., dog barking) within the human audible frequency range. The relative amplitudes of each cue were calibrated so as to be of equal perceived intensity at the auditory detection threshold volume.

#### Closed-Loop Targeted Memory Reactivation (CL-TMR)

We developed a first-of-its-kind closed-loop system for delivery of auditory cues to drive TMR. The system times the delivery of auditory cues to DUPTs. Our CL-TMR system leverages two parallel signal processing pipelines which perform online detection of DUPT events and identification of NREM sleep state. Both pipelines were implemented using the OpenViBE open source software platform^1^. This software has the benefits of interfacing with multiple EEG recording systems, including the BrainAmp DC system used for the present study, as well as an extensive library of signal processing tools for real-time analysis of signals.

The most critical aspect to the accuracy of the system is the identification of NREM2/NREM3 sleep. We developed a novel approach to doing real-time sleep staging based on the recommended biomarkers for each stage of sleep from the American Association of Sleep Medicine (Iber et al., 2007). All NREM stages of sleep are computed from a ratio of spectral power in the Delta, Alpha, and Gamma (DAG) ranges. Specifically, DAG refers to the ratio of Frontal Delta to Occipital Alpha times Global Gamma. *Frontal Delta* corresponds to the spectral power in seven frontal channels (Fp1, Fp2, Fz, F3, F4, F7, F8). *Occipital Alpha* refers to the spectral amplitude in the frequency range 8–12 Hz at the Oz, O1, and O2 electrode positions. *Global Gamma* refers to the spectral amplitude in the frequency range 25–50 Hz at all 32 electrode positions.

The DAG metric is computed over non-overlapping 5-s epochs and thresholded to produce the various stages of sleep between 0 (wake) and 3 (NREM3). In practice we have found the following thresholds work well in our system; wake < 0.03 (NREM1) < 0.05 (NREM2) < 0.25 (NREM3). Algorithm parameters were developed and optimized using data from Day 1 screening naps and Day 2 and Day 3 naps in control participants (*N* = 51 naps from 32 control participants). An experienced sleep researcher scored the nap data and the manually scored records were used as a reference to guide iterative adjustments of the thresholds of sleep stage identification in order to maximize the system’s performance across participants. These identified sleep stages were then used to gate the delivery of auditory cues with coincident detection of DUPT events.

The SO is believed to originate in the frontal cortices and coordinates the processes of memory consolidation in the two-stage model (Buzsaki, 1998; Marshall and Born, 2007; Diekelmann and Born, 2010). Thus we employed a commonly used and relatively simple detector for SO events by averaging frontal channels (Fp1, Fp2, Fz, F3, F4, F7, F8) and looking for instances where the mean potential crosses -80 μV (Ngo et al., 2013, 2015). To safeguard against slow drift, an additional check is employed during a detection that the signal originated from a positive potential at some point during the previous 400 ms. After a SO is detected in NREM2/3 sleep, a cue is played. The sounds are concurrently played through the StimTrak device and directly into a channel on the auxiliary BrainAmp ExG amplifier with a low-frequency carrier wave to easily identify individual cues and their timing relative to the ongoing neurophysiological data.

Our current sleep staging pipeline does not identify rapid eye movement sleep (REM). However, the occurrence of DUPT events during REM is very rare resulting in very few false positives. The current false positive rate for DUPT detection is under 0.04 and occurs primarily with arousal events when participants wake from NREM2/NREM3 sleep.

### Procedure

**FIGURE 2:**
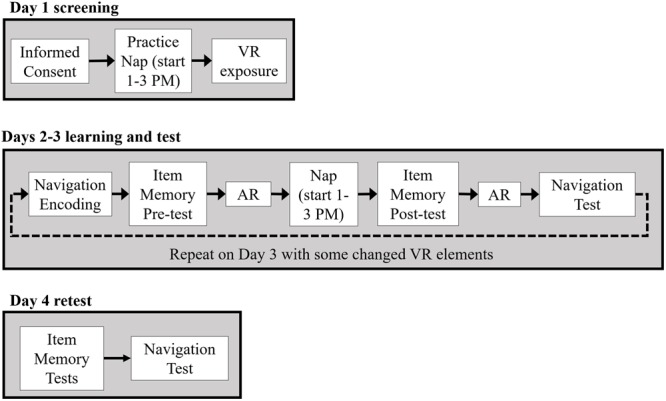
After an initial screening day (Day 1), Days 2 and 3 followed the same overall procedure with participants fully instrumented. Participants first trained on wayfinding tasks in the VR environment. Following encoding, a declarative item memory pre-test was administered followed by the first awake replay (AR) session. During a ∼90 min nap, auditory cues were presented to the CL-TMR participants whereas control participants napped without cue delivery. After the nap, participants took a declarative item-memory post-test, underwent a second AR session, and took a navigation test on a subset of routes encountered during encoding. Participants returned the following day for Day 3 and underwent the same procedure except some elements were changed in the VR training task and test. On Day 4, participants were not fully instrumented while taking declarative memory tests and a final navigation test.

**Figure 2** provides a schematic of the experimental procedure. All participants came for an initial screening day, Day 1, during which they gave informed consent and became acquainted with navigating in a VR environment. A subset of early participants (*N* = 9 controls, *N* = 7 CL-TMR) was also instrumented with EEG, EOG, and ECG electrodes as described previously and attempted a 90-min nap. The screening day allowed the researchers to determine whether the participants would be able to nap, fully instrumented, in an unfamiliar place and would be able to tolerate the VR headset for about 15 min or more at a time, as nausea is a common side effect. Those who passed the VR and nap screening were scheduled to continue the experiment. Those who were ineligible were compensated for their time and dismissed. Ultimately, since no differences were observed between those who took a screening day nap and those who did not, we stopped requiring the screening nap and subsequent participants only completed VR screening on Day 1.

All eligible participants came in for three consecutive days of experimental sessions. On Day 2, participants were first instrumented with EEG (the EOG electrodes were applied just before the nap, as it would interfere with the placement of the VR headset). Vibratory feedback coincident with the sounds in the VR environment was delivered by a haptic gaming vest (Kor-FX, Immerz, Inc., Cambridge, MA, United States). The purpose of the vest was to increase the salience of the auditory cues at the district boundaries.

Once instrumented, the participant began the training phase of the navigation task. Participants were instructed to pay attention to the sounds and location when crossing the district borders of the map. At the start of each of the 24 distinct routes, participants were informed of the destination on the virtual cell phone. During navigation, the “non-optimal route alert” occurred at decision points if the participant was not on the optimal (shortest) path. During training, routes were organized into blocks of contiguous routes to promote learning of the global organization of the environment; for example, if one route ended at the coffee shop, the next route would begin at the coffee shop. The 24 routes were divided into three groups of seven to nine routes each in order to give participants breaks from the headset, but participants could take as many breaks as they wished during training to prevent nausea.

Following training, participants took a 20-question declarative memory pre-test which probed for knowledge about the learned VR environment before any intervention occurred. Ten questions were short-answer (cued recall) and the other 10 were multiple-choice (recognition). For the purpose of the test, a “landmark” was defined as a specific location one navigated to or started from on any route during the training phase. The short-answer questions took the following forms: (1) participants were shown a picture of a landmark and asked to name the two closest landmarks to the pictured one; (2) participants were told to imagine themselves standing in a pictured location and asked to name the closest landmark to the left, right, or behind them; (3) participants were again asked to imagine standing in a pictured location, and asked to name a landmark that they could see if they were to turn in that spot; and (4) participants were shown two different landmarks and asked to name a landmark that would be passed if traveling along the optimal route from the first to the second landmark. For the multiple-choice questions, (1) participants were shown an image of the surroundings at a border crossing and asked to select the auditory cue that was heard; (2) participants were shown an image of a landmark and asked to select the direction (straight, left, right, backward) one must travel to get to a boundary landmark or a local landmark; and (3) participants were asked to choose the landmark that does not belong in the same district as the others.

Prior to consolidation with sleep, memories are thought to be labile and prone to interference. Memory reactivation after learning, during waking periods, has been shown to reduce subsequent recall (Diekelmann et al., 2011). However, one study has shown that TMR during wakefulness can lead to memory improvements (Oudiette and Paller, 2013). We tested whether our CL-TMR intervention is robust to interference by re-exposing participants to the auditory cues either during a concurrent interference task or a period of quiet wakefulness after the initial training interval. The 18 cues were repeated five times in the same order with a jittered interstimulus interval varying from 3 to 6 s. Participants were asked to either remain quietly awake (no interference), or complete a competing auditory or visual task at the same time. Participant assignment to each of these conditions was counterbalanced within each cohort. Auditory interference consisted of listening to a compilation of famous movie themes composed by John Williams that lasted approximately the same amount of time as the five cycles of auditory cues. Visual interference consisted of playing the game Tetris while listening to the cues. Both cohorts were exposed to the interference tasks and counterbalanced within each cohort to ensure equal numbers completed each interference condition.

Following the awake replay session, participants were required to take a nap. Electrodes were placed at the corners of the eyes for the EOG recording and the haptic vest was removed. Participants were assigned to either a control condition in which they napped without any intervention, or a CL-TMR condition in which auditory cues were delivered during the nap using the closed-loop system described above. If they were in the CL-TMR cohort, participants wore soft, flat earphones that rested over the outer ear (a confound that is elaborated on in the section “Discussion”) and the volume was calibrated prior to the nap using an adaptive staircase procedure until a volume level just above auditory threshold was reached. All participants started sleeping between 1 to 3 PM and were allowed to sleep for ∼90 min. During the nap, the volume was reduced if cues decreased the DAG measure (indicating arousal). Conversely, if the cues did not decrease the DAG measure and no spindle response was observed, the volume was increased. Participants were awakened after about 90 min from time they fell asleep. A mandatory break of at least 30 min after the nap occurred before the final tasks in order to minimize the effects of sleep inertia. Participants used this time to complete a demographics questionnaire, the Big Five Inventory (John et al., 1991), and the State-Trait Anxiety Inventory Y-2 (Spielberger et al., 1983).

Following the sleep inertia break, participants were given a declarative memory post-test with the same format as described for the pre-test declarative memory test but with different questions. The order of the two declarative memory tests was counterbalanced. After the test, participants underwent a second session where auditory cues were delivered in the absence of an interference task. Responses to cues were collected to try and assess any impact of the nap and intervention on awake memory replay, however, reliable and specific cue-evoked responses were not able to be identified so these analyses have been omitted in this manuscript. Finally, a navigation test in the same environment on a subset of six routes was given. Non-optimal route alerts were turned off, and speed and accuracy were recorded. Two different route sets (of 6) were tested (and counterbalanced) across participants at each post-testing interval. Thus, 12 routes were tested at Day 2 and Day 3 in each cohort, with the remaining 12 tested on Day 4. This was done to balance some of the variability in route difficulty and location within the environment across each day. At the end of the experimental session, participants were given an actigraphy device (Philips, Andover, MA, United States) to monitor overnight sleep. If an actigraphy device was not available, participants were asked to report the number of hours slept the previous night.

This procedure was repeated for Day 3, with the only difference being that roadblocks were placed in the environment such that for 13 of the 24 routes, the participant had to identify a new optimal route. The same roadblocks were also present in the VR environment for testing on Day 3. The testing route lists on Days 2 and 3 were counterbalanced across participants using two sets of six routes to better sample the environment and maintain equivalent levels of difficulty. Thus within a cohort each testing day had 12 total routes that were tested (but only 6 in each participant for Days 3 and 4).

On Day 4, participants were not instrumented. They took declarative memory tests designed to probe knowledge of the spatial layout of the landmarks in relation to the districts. On the first test, they were shown a picture of a representative building from each of the six districts and were asked to mark the location of that specific building on a map like the one shown on the virtual cell phone. On the second test, participants were given a list of all landmarks (places they could have navigated to or from during the learning and test phases) and asked to mark which district each landmark belonged in. Finally, participants were tested inside the VR for their ability to navigate the remaining 12 routes in the map without roadblocks. To avoid test/re-test confounds, in each participant the 24 routes were divided such that no route is tested twice (6 on Day 2, 6 on Day 3, and 12 on Day 4).

### Statistical Analyses

In all cases where non-parametric tests were used, it is due to a lack of normality in the sample. Behavioral results were analyzed with non-parametric, two-tailed Mann–Whitney *U* tests to test our a priori hypothesis that CL-TMR would improve navigation ability. The nap data were visually scored by an experienced sleep researcher, and sleep parameters were analyzed with two-tailed Mann–Whitney *U* tests and the Bayesian Information Criterion as described in Wagenmakers (2007). Statistics on spindle energy were performed using a two-sample two-tailed *t*-test. Because no meaningful results were found involving the declarative memory tests nor the impact of sensory interference, they will not be discussed further.

With the exception of the online system developed for CL-TMR all other analyses were performed offline in MATLAB (MathWorks, Natick, MA, United States) using the BioSig toolbox^2^ and EEGlab toolboxes^3^ (Delorme and Makeig, 2004). Following collection, EEG data was preprocessed as follows. All signals were re-referenced to the linked mastoids and high-pass filtered at 0.2 Hz. Spindle energy was quantified by bandpass filtering the data in the slow (9–12 Hz) or fast (12–15 Hz) spindle range, *z*-scoring the data to reduce across-participant differences in signal magnitude, and summing the envelope of the magnitude of the signal to get the area under the curve during a 1 s epoch starting with the onset of the up state (positive potential) following a DUPT. Analyses requiring adjustment for false discovery rate in hypothesis testing were conducted using the approach described in Benjamini and Hochberg (2000).

## Results

**FIGURE 3:**
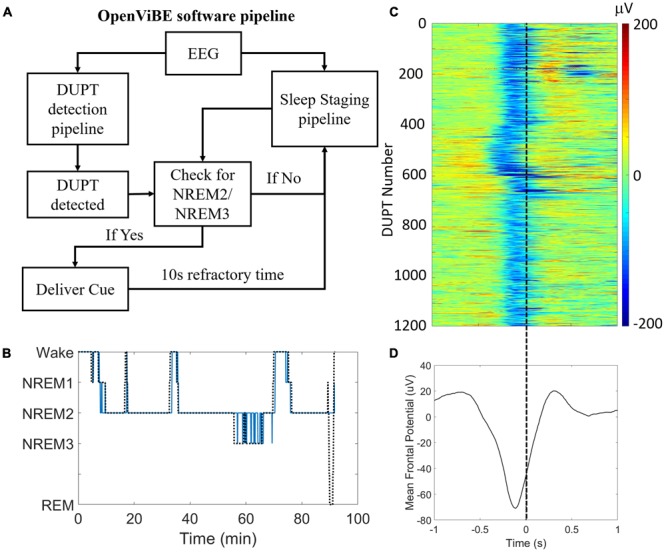
Our closed-loop system delivers consistent and accurate stimulation during DUPTs. **(A)** A block flow schematic of the control-logic in our OpenViBE software. **(B)** Results of our online sleep scoring in a single subject’s nap session. Sleep staging serves as a gate for identifying valid DUPT events and thus the most important criterion for accuracy is the correct identification of NREM2 and/or NREM3 sleep. **(C)** Across subjects’ performance of our closed-loop system for identifying DUPT events. The event-related potentials (ERPs) plot shows over 1100 individual DUPTs taken from our CL-TMR participants where a cue was delivered. Time 0 indicates the time of actual cue delivery which is consistently just before the time of transition to the up-state. **(D)** The grand average ERP in the plot below shows the mean time of stimulus delivery. Note that because the ERPs are locked to the time of the actual cue delivery rather than the detection event (-80 μV crossing), the minimum negative potential of the mean ERP fails to cross -80 μV.

**Figure 3A** shows the operation and logic of our closed-loop TMR approach. **Figure 3B** shows the results of our automated sleep-staging algorithm in a single subject’s nap session. In the nap shown, NREM2/NREM3 sleep was correctly identified against visual scoring with a recall of >0.95 [true positives/(true positives + false negatives)] and a precision of >0.93 [true positives/(true positives + false positives)]. Across participants, the recall and precision of the system were 0.89 and 0.85, respectively.

The detection of DUPTs and latency of cue delivery is shown in **Figure 3C** across Day 2 naps within our CL-TMR cohort. The plot shows the mean potential across the frontal (Fp1, Fp2, Fz, F3, F7, F4, F8) electrodes for each DUPT which triggered a cue (*n* = 1190). The event related potentials (ERPs) demonstrate a high degree of consistency in the timing across participants. The mean ERP is shown in **Figure 3D**. The plot shows that the cues are delivered with a mean latency of 238 ± 42 ms from the time of the detection event (-80 μV crossing). This timing precedes the transition to the up-state by >100 ms on average. Note that because the ERPs are locked to the time of the actual cue delivery which is jittered with respect to the (-80 μV) detection event, the minimum negative potential of the mean ERP in the plot fails to cross -80 μV.

### CL-TMR Impact on Sleep Architecture

To investigate the impact of the intervention on the sleep architecture, all recordings were visually scored using 30-s epochs according to the AASM rules (Iber et al., 2007). Standard sleep parameters such as the total sleep time, the time and the percentage of time spent on each sleep stage (NREM1, NREM2, NREM3, and REM), the minutes of wake after sleep onset (WASO), the sleep efficiency (the ratio between the time spent asleep and the time spent in bed), and the sleep onset latency were extracted. These values are presented in **Tables 2, 3**. Mann–Whitney *U* tests showed no group differences for any parameters either at Day 2 or at Day 3. Two parameters, the total sleep time and sleep efficiency, were trending toward significance on Day 2, but in both cases CL-TMR subjects showed better sleep compared to the controls. To support these null results, we calculated the approximate Bayes Factor (BF_10_) through the Bayesian Information Criterion (BIC), following the procedure described in Wagenmakers (2007) and Jarosz and Wiley (2014). On both days, for most of the parameters we observe a BF_10_ below 1, strongly supporting the absence of significant differences. Overall these results indicate that the intervention did not negatively impact sleep architecture. Additionally, we found no significant correlation between the time spent in NREM2 (*r* = -0.11, *p* = 0.70), NREM3 (*r* = -0.15, *p* = 0.59), or total sleep time (*r* = 0.09, *p* = 0.74) and performance during Day 2 in participants receiving the CL-TMR intervention, suggesting that performance differences were not due to differences in sleep architecture. Results comparing sleep time to performance during Day 3 and across all testing intervals were similarly uncorrelated.

**Table 2:** Sleep parameter comparison between control and CL-TMR participants for Day 2.

Sleep Parameter	Controls (*N* = 20)	CL-TMR (*N* = 17)	*U*	*Z*	*p*	Mean difference	*d*	BF_10_
TST (min)	66.05 19.88	78.62 12.23	106	–1.95	0.05	–12.57	–0.75	2.22
SOL (min)	8.80 5.26	7.21 6.55	122	1.46	0.14	1.59	0.27	0.42
NREM1 (min)	9.25 4.34	9.12 6.59	146.5	0.72	0.47	0.13	0.02	0.32
NREM2 (min)	41.00 17.50	47.44 19.65	137	–1.01	0.31	–6.44	–0.35	0.49
NREM3 (min)	12.58 11.02	20.06 15.59	121.5	–1.48	0.14	–7.48	–0.56	0.98
REM (min)	3.23 6.19	2.00 4.81	169	–0.03	0.98	1.23	0.22	0.38
NREM1 (%)	16.69 13.29	12.10 8.84	127.5	1.30	0.20	4.59	0.40	0.57
NREM2 (%)	61.09 15.73	59.09 20.18	167.5	0.08	0.94	2.00	0.11	0.33
NREM3 (%)	18.26 13.99	26.27 19.19	130.5	–1.20	0.23	–8.01	–0.48	0.73
REM (%)	3.95 7.37	2.55 6.06	168	–0.06	0.95	1.40	0.21	0.37
WASO (min)	13.30 16.21	7.32 5.55	129.5	1.23	0.22	5.98	0.48	0.72
SE (%)	74.65 17.33	83.88 11.78	110	–1.83	0.07	–9.23	–0.61	1.20

*BF_*10*_, Bayesian Factor; d, Cohen’s d; p, p-value; REM, rapid eye movement sleep; TST, total sleep time; SOL, sleep onset latency; SE, sleep efficiency; U, Mann–Whitney U-statistic; WASO, wake after sleep onset; Z, Mann–Whitney Z-statistic*.

**Table 3:** Sleep parameter comparison between control and CL-TMR participants for Day 3.

Sleep Parameter	Controls (*N* = 20)	CL-TMR (*N* = 17)	*U*	*Z*	*p*	Mean difference	*d*	BF_10_
TST (min)	66.05 18.92	71.21 21.37	132	–1.16	0.25	–5.16	–0.26	0.41
SOL (min)	12.20 17.25	5.58 3.21	120.5	1.51	0.13	–1.48	–0.21	0.81
NREM1 (min)	10.55 6.63	12.03 7.70	153	–0.52	0.60	–1.06	–0.06	0.37
NREM2 (min)	39.83 15.35	40.88 17.69	164	–0.18	0.85	–4.99	–0.37	0.32
NREM3 (min)	10.60 11.55	15.95 15.35	133	–1.13	0.26	–7.48	–0.56	0.52
REM (min)	5.08 9.11	2.71 6.09	139	0.94	0.34	2.37	0.30	0.44
NREM1 (%)	17.11 12.54	20.61 17.21	160.5	–0.29	0.77	–3.50	–0.24	0.39
NREM2 (%)	59.98 15.96	56.78 15.93	149.5	0.62	0.53	3.20	0.20	0.37
NREM3 (%)	15.95 15.35	19.41 18.82	149.5	–0.62	0.53	–3.46	–0.20	0.37
REM (%)	6.19 10.53	3.21 6.90	139	0.94	0.34	2.98	0.33	0.47
WASO (min)	11.83 13.43	14.91 20.18	147	0.70	0.48	–3.09	–0.18	0.36
SE (%)	77.86 18.92	77.60 22.42	122.5	–1.45	0.15	–4.72	–0.23	0.39

*BF_*10*_, Bayesian Factor; d, Cohen’s d; p = p-value; REM, rapid eye movement sleep; TST, total sleep time; SOL, sleep onset latency; SE, sleep efficiency; U, Mann–Whitney U-statistic; WASO, wake after sleep onset; Z, Mann–Whitney Z-statistic.*

### CL-TMR Improves Navigation Efficiency

**FIGURE 4:**
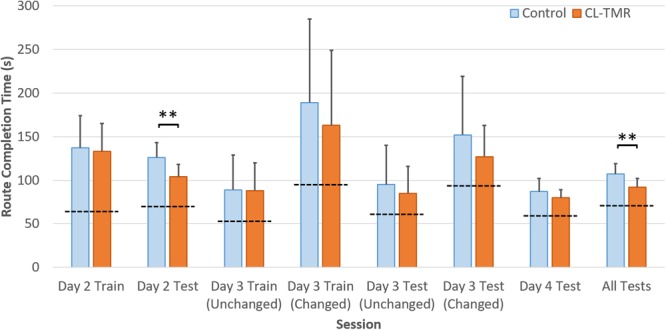
The average navigation times across sessions by cohort in **Table 4** are shown. Error bars are SDs. The dotted lines indicate the optimal navigation time necessary to complete all the routes in a given session. ^∗∗^Indicates statistical significance between cohort performance in that interval (Mann–Whitney *U* test, *p* < 0.01).

Because the variances across all of the route times were extremely high due to highly variable route lengths and therefore navigation times, the data were normed before statistical analysis. For every participant, each route time was divided by its across-participants mean. Analyses were performed on these normed values. **Table 4** shows the mean non-normed route times for each cohort at each training and testing interval to show the relative gains in absolute time. **Figure 4** illustrates the behavioral results reported in **Table 4**.

**Table 4:** Participants who received CL-TMR show reduced navigation time.

	Day 2 train (trials = 24)	Day 2 test (trials = 6)	Day 3 train (trials = 11/13)	Day 3 test (trials = 3/3)	Day 4 test (trials = 12)	All test (trials = 24)
Control (*N* = 20)	137 (37)	126 (17)	89 (40)/189 (96)	95 (45)/152 (67)	87 (15)	107 (12)
CL-TMR (*N* = 17)	133 (32)	104 (14)^∗∗^	88 (32)/163 (86)	85 (31)/127 (36)	80 (9)	92 (10)^∗∗^
Optimal navigator	64	70	53/95	61/93	59	71

*Mean navigation time for each training and testing interval is shown in seconds, with ±standard deviation in parentheses. The All Test condition refers to the mean of all testing intervals across days 2–4. The Optimal Navigator refers to the minimal time required to complete all trials in that interval if one took the ideal path. ^∗∗^Indicates statistical significance between cohort performance in that interval (Mann–Whitney U test, p < 0.01).*

Both groups show similar training time on Day 2, however, the CL-TMR group shows significantly reduced testing time following the nap (two-tailed Mann–Whitney *U* test: *N*1 = *N*2 = 12 routes, *p* = 0.003, Cohen’s *d* = 1.41). Although not statistically significant, the CL-TMR group also shows enhanced performance in terms of reduced navigation times at testing on Days 3 and 4 in comparison to controls. When considering testing across all 3 days, the CL-TMR cohort exhibits significantly improved performance, (two-tailed Mann–Whitney *U* test: *N*1 = *N*2 = 24 routes, *p* = 0.0002, Cohen’s *d* = 1.36). Also notable is the reduced variability among CL-TMR participants relative to controls. This is especially visible in the Day 3 test and suggests that the intervention is delivering a consistent benefit and normalizing learning. The Day 3 training and testing intervals are subdivided into trials where the optimal path remained unchanged and those where newly placed impediments required the participant to identify a different optimal path. These changes in the environment naturally increase training and testing times on these trials for both cohorts; however, the CL-TMR group shows nominally reduced navigation time on the changed routes at training and testing compared to controls, suggesting that they may be more capable of manipulating a cognitive map to quickly identify new, more optimal routes. Higher variability in Day 3 performance on changed routes as well as a limited number of testing trials in each condition likely contributes to a lack of significance in this interval. By Day 4, control participants have made up much of the difference that is observed in Day 2. Thus, the strongest benefits of CL-TMR are observed in the immediate interval following learning.

### CL-TMR Increases Fast Spindle Activity

**FIGURE 5:**
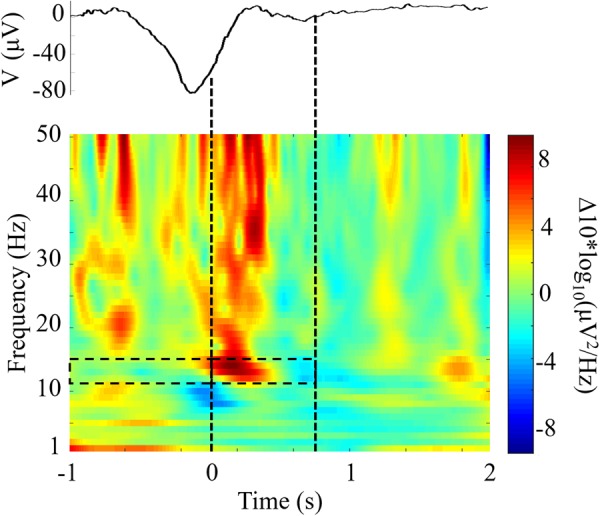
Closed-loop targeted memory reactivation (CL-TMR) evokes a robust increase in spindle frequency power spectral density. The time-frequency plot shows the difference between the mean response in TMR participants (*N* = 1190 epochs) and the response at the same time point in control participants (*N* = 1592 epochs) across electrode locations Fp1 and Fp2. Time 0 indicates the median time of cue presentation, 238 ms after the -80 μV crossing. Statistical significance was tested in the 1-s pre and post cue intervals in the fast spindle band (12–15 Hz) across participants. The mean event related potential over electrodes Fp1 and Fp2 is shown above the time-frequency plot. Dashed lines indicate the median window of cue delivery (length 700 ms).

We compared the responses to TMR cues with the same DUPT-locked events in control participants that received no stimulation to investigate the impact of CL-TMR. **Figure 5** shows the differences in mean power spectral density between DUPTs occurring in control participants over the mean of channels Fp1 and Fp2 and the set of DUPTs in CL-TMR participants that received auditory cues. The frontopolar channels were chosen here since (as shown in the next section) these channels show a significant and representative increase in fast spindle energy when comparing controls to TMR participants. Power spectral density was computed from data processed with a continuous wavelet transform (Morlet wavelet) in the frequencies from 1 to 50 Hz and over the time interval from 1 s before the median time of cue delivery to 2 s after cue delivery. **Figure 5** shows that the largest spectral differences exist in the fast spindle band (12–15 Hz). These differences are most pronounced in the first 500 ms following the cue onset. More modest differences can also be seen in the theta band and larger increases are observed in the low gamma band. Mean differences in the fast spindle band (12–15 Hz) during cue delivery were statistically significant (two-tailed independent samples *t*-test, *p* < 0.01) across participants but were non-significant in the 1-s window preceding the cue (two-tailed independent samples *t*-test, *p* = 0.76).

### Topography of Spindle Activity

The spatial distribution of nested spindle activity may vary depending on the type of memories being consolidated (Yordanova et al., 2017). We investigated topographical differences in DUPT-locked spindle energy between control participants’ naps on Days 2 and 3 of our protocol (following our learning task) and naps taken on their screening day (Day 1) when no learning task was administered. Only control participants were compared in this way to avoid conflating the effects of the intervention with those from task-specific learning.

**FIGURE 6:**
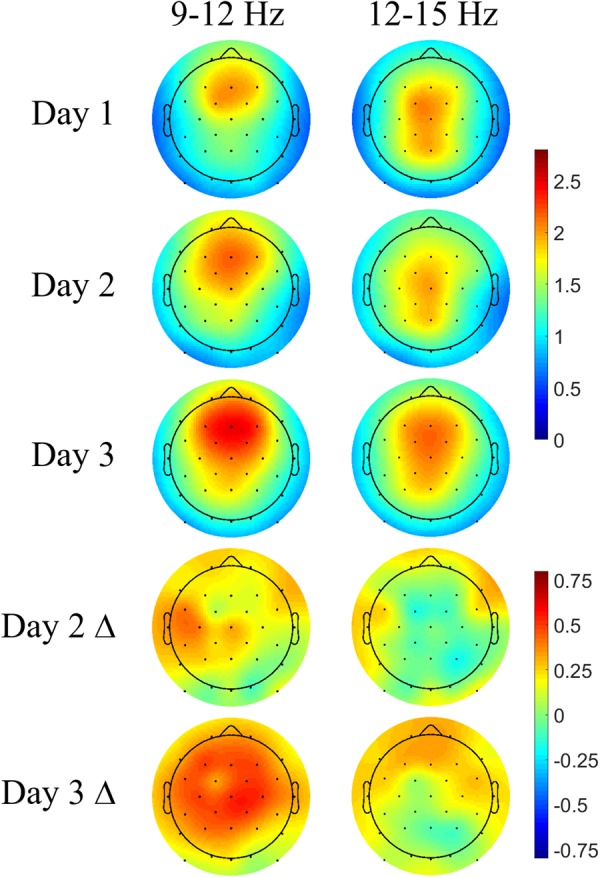
Navigation learning increases spindle energy in controls. Topoplots show the mean DUPT-locked spindle energy (*z*-scored) for each day and the change between Days 1 and 2 (Day 2 Δ) and Days 1 and 3 (Day 3 Δ). Slow spindles (9–12 Hz) show a more frontal topography across days. Increases in Day 2 are small and localized to frontal and centroparietal electrodes. Day 3 differences are larger and more widespread covering almost the entire head. Differences in fast spindle topography are mostly localized to frontal electrodes and are stronger on Day 3. Color bars indicate units of standard deviation in spindle amplitude.

Each participant’s mean spindle energy in the fast (12–15 Hz) and slow bands (9–12 Hz) was computed over a 1-s interval during the subsequent up state following DUPTs. The topoplots in **Figure 6** show typical spatial bias for slow spindles to be more anterior while fast spindles are more central and posterior across all 3 days (Zeitlhofer et al., 1997). Overall stronger spindle energy is observed in Day 3 compared with Day 2, perhaps indicating a benefit of more practice and learning on the task. Differences in the spindle energy topography between Days 2 and 1 (Day 2 Δ) show no significant differences, but do show nominal increases in spindle energy in frontal and some centroparietal electrodes for slow spindles. Similarly, the differences in spindle energy topography between Days 3 and 1 (Day 3 Δ) are not statistically significant, but do show widespread increases in slow spindle energy, and more focal increases in fast spindle energy in frontal electrodes. We next compared the mean spindle energy in participants that received CL-TMR to investigate evoked differences in spindle topography.

**FIGURE 7:**
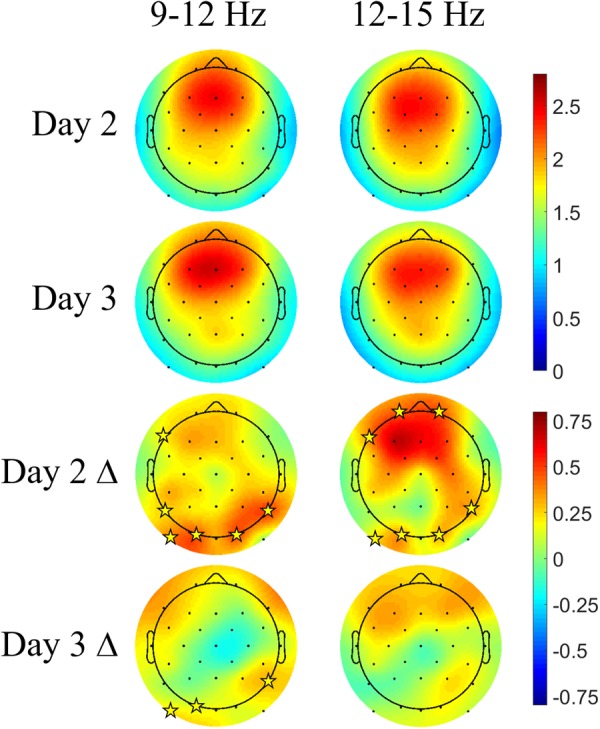
Closed-loop targeted memory reactivation increases spindle energy compared with normal learning. Topoplots show the mean DUPT-locked spindle energy (*z*-scored) for Days 2 and 3, and the difference between Days 2 and 3 of CL-TMR participants and the same day topoplots in controls (Day 2 Δ and Day 3 Δ). Compared with controls, participants receiving CL-TMR show significantly higher spindle energy on Day 2 at frontal and right parietal sites. Stars indicate electrode sites with significant differences after adjustment for false discovery rate (*p* < 0.05). Color bars indicate units of standard deviation in spindle amplitude.

**Figure 7** shows the Days 2 and 3 mean spindle energy topoplots for our CL-TMR participants as well as the difference between these plots and the control topoplots in **Figure 6** of the same day. Overall, CL-TMR can be seen to drive increases in spindle energy on both days, however, these differences are largest on Day 2 owing primarily to the weaker energy observed in controls on Day 2. Day 2 differences are largest in one cluster of frontal and another cluster of occipitoparietal electrodes for fast spindles and only in occipital and right parietal electrodes for slow spindles. Day 3 shows smaller increases in fast and slow spindle energy in mostly frontal electrodes with slow spindles also showing some significant increases in right parietal and left occipital sites. Differences at starred electrode locations are significant after adjustment for false discovery rate (see **Table 5**). However, only mean fast spindle energy in the starred frontal cluster (Fp1, Fp2, F7) is correlated with Day 2 navigation performance (all participants, *r* = 0.41, *p* = 0.02). There were no significant correlations between spindle energy in the occipitoparietal cluster (P8, PO9, O1, O2) and behavior at any interval. Similarly, there were no significant correlations between slow spindle energy in either cluster and navigation performance.

**Table 5:** Electrode locations and statistics corresponding to **Figure 6**.

	Slow (9–12 Hz)	*p*	*t*	Fast (12–15 Hz)	*p*	*t*
CL-TMR	F7	0.008	2.6	Fp1	0.0006	3.9
Day 2 Δ	P7	0.002	3.3	Fp2	0.015	2.6
	P8	0.005	2.8	F7	0.003	3.4
	PO9	0.0005	3.7	P8	0.004	3.3
	O1	0.0003	3.9	PO9	0.002	3.5
	O2	0.0015	3.4	O1	0.0004	4.1
				O2	0.007	3.0
Day 3 Δ	P8	0.0012	3.6			
	PO9	0.0018	3.5			
	O1	0.008	2.9			

*P-values from two sample t-tests corresponding to the starred locations indicating significant differences in **Figure 6**. For CL-TMR participants, Days 2 and 3 Δ refer to the difference between the control and CL-TMR cohorts in spindle energy. Significant channels have been adjusted for false discovery rate (df = 30, mastoids excluded)*.

## Discussion

In the current study, we developed a system that allowed closed-loop delivery of TMR protocols during sleep. CL-TMR enhanced navigation skills and increased spindle energy compared with non-stimulated sleep. Our results showed that sleeping with the closed-loop TMR (CL-TMR) system is feasible, can be successfully used during a daytime nap, and, importantly, did not negatively impact sleep architecture. Previous studies with TMR have demonstrated its success in a range of reasonably well-controlled laboratory tasks (e.g., Rasch et al., 2007; Schreiner and Rasch, 2014). Although our task was complex and allowed for less control over certain variables, our navigation task has a relatively high level of ecological validity. It was conducted within a large-scale urban environment in VR that resembled the experience of navigating through the downtown area of a city and was characterized by congruent sensory stimulation (e.g., the environmental sounds). While all the participants learned how to navigate through the city to reach specific landmarks, participants receiving CL-TMR showed greater improvement (i.e., more time-efficient navigation) in the task after one nap with auditory stimulation compared to the controls. In our study, behavioral differences and the accompanying neurophysiological impact of intervention were strongest after the period of initial learning, and these effects were diminished in subsequent days of testing. We propose that the reduced efficacy of the intervention observed in Days 3 and 4 is most likely due to the benefits of overnight sleep and repeated practice on the task which are equally realized by both cohorts.

Using the large magnitude negative potentials (-80 μV) to reliably detect SOs during NREM3 has been demonstrated previously (Ngo et al., 2013, 2015). Here we have developed a reliable approach to identifying both NREM2/3 sleep, which also enables the detection of k-complexes. This is particularly important when one wants to leverage naps with CL-TMR since participants do not reliably spend a significant amount of time in NREM3 during a 90-min napping session. While this approach results in a smaller number of opportunities to deliver CL-TMR, positive effects have been shown with as few as a single presentation each of 25 unique cues (Rudoy et al., 2009; Creery et al., 2015; Batterink et al., 2016).

We targeted the delivery of the cues during the transition from cortical down-states to up-states as determined by the crossing from negative to positive potential of the SO or k-complex. Our system consistently delivered the sensory stimulation with a median latency of 238 ms (*SD* = ±54 ms) or at the ∼300° phase of the slow oscillation and on average 100 ms prior to the zero crossing into the next cortical up-state. A recent study into the relationship between the phase of the slow oscillation at the time of cue delivery and its impact on memory found that this phase bin (270–360°) represented a relatively suboptimal time for memory enhancement in a cued spatial association task, whereas the optimal phase bin was 180–270° degrees (Batterink et al., 2016). The positive effect observed in our study could be due to task differences, as our task was a spatial navigation task in VR in a 3D virtual environment whereas their subjects were required to memorize spatial locations of objects on a 2D grid. Another possibility is that, due to the variability in the timing of cue delivery, a subset of cues that were presented in our study fell into the optimal phase bin suggested by Batterink et al. (2016) and may have been sufficient to drive improvements. Still another possibility is that due to the limited resolution of the bins in the Batterink study, the true optimal phase bin is somewhere in the middle and closer to the timing used in this study. A recent non-peer reviewed study has shown positive results with TMR using timing similar to what was implemented in this study (Göldi et al., unpublished).

Neurophysiologically, the CL-TMR intervention increased fast spindle activity (12–15 Hz) locked to DUPTs (see **Figure 7**). This result is in line with recent studies in which cues delivered after the negative peak of the SO led to increases in fast spindle power (Ngo et al., 2013, 2015; Leminen et al., 2017). We observed increases in fast spindle energy for the CL-TMR cohort compared to controls in frontal, parietal, and occipital regions on Day 2. Although the increase in parietal regions is consistent with other findings (Zeitlhofer et al., 1997), the increase in fast spindle activity at frontal locations is a less commonly reported phenomenon. However, one study demonstrated that regions including the orbitofrontal and medial prefrontal cortices as well as the hippocampus were preferentially active in response to fast spindles (Schabus et al., 2007). Similarly, another study indicated that fast spindles during NREM2 may enhance functional connectivity between the hippocampus and the neocortex, including the medial prefrontal cortices (Andrade et al., 2011). Fast spindle activity in the prefrontal cortex has been positively correlated with subsequent episodic associative learning ability, and further, fast spindle activity in the left prefrontal cortex was positively correlated with subsequent changes in learning ability (Mander et al., 2011). In this study, we have similarly shown that only fast spindle activity in frontal electrode sites correlates with navigation performance. Taken together, these results are consistent with the idea that fast spindles support the transfer of reactivated memories from the hippocampus to the cortex (Walker, 2009; Rasch and Born, 2013). Although there were statistically significant differences in spindle energy at occipitoparietal locations, the overall mean energy in these locations is low. Thus the large increases seen in those areas may be due more to a floor effect in the control comparison. Nevertheless, the observed increases in spindle energy in right posterior parietal sites following navigation learning is interesting given that these types of tasks have been shown to engage these areas of the brain (Hartley et al., 2003).

Since our approach targets large amplitude slow oscillations in both NREM2 and NREM3 sleep it is possible that there are differences in the response to cues delivered during k-complexes vs. slow oscillations in SWS, and that these may explain some of the behavioral variance. However, analysis of the spectral power density in the spindle band found no significant differences between cues delivered during NREM2 and NREM3 on any channel (individual time-frequency plots not shown, all *p*-values > 0.13), and no correlation was revealed between time spent in either sleep stage and performance. One caveat to this is that not all participants spent a significant amount of time in NREM3 due to the duration of the nap. Applying this approach during overnight sleep would be useful in elucidating the relative contributions of cues delivered during NREM2 vs. NREM3 sleep.

A potential confound in the present study is that the control participants did not wear the soft earphones during the nap, whereas the CL-TMR participants did. The objective in doing this was to safeguard against impediments to sleep quality in our control participants. During Day 2 we observed an increase in total sleep time in our CL-TMR participants and non-significant nominal differences in other sleep parameters. The possibility that these effects were driven only by wearing earphones cannot be completely ruled out. However, no correlation was found between NREM2, NREM3 or total sleep time, and our primary behavioral effect of navigation time, making this a less likely explanation for the observed improvements. Another limitation of the present study is that it is unclear whether the specificity of the auditory cues to the navigation environment was a crucial factor in the observed enhanced spindle activity and navigation performance, however, multiple previous studies have suggested that specificity may be important in this regard. Odor context specificity during sleep appears to be required for increased fast spindle power and improved performance on a visuospatial task (Rasch et al., 2007; Rihm et al., 2014). Similarly, cueing items during sleep with sounds associated with encoding strengthens spatial memories in comparison to uncued items (Rudoy et al., 2009; Creery et al., 2015). Furthermore, EEG responses to a sound that was not associated with encoding were different from the responses to the sounds that were associated with learned items. An imaging study revealed increased parahippocampal activation in response to auditory TMR cues vs. control sounds, and post-sleep retention was positively correlated with TMR cue-related activity (van Dongen et al., 2012). Together, these studies indicate that associating sounds to learning material during encoding causes them to be processed differently, even during sleep, and possibly is critical for selectively strengthening memory. A related question is whether cue congruency with the learning environment or stimuli is a necessary factor in enhancing spindle activity and behavioral performance. Rudoy et al. (2009) and Creery et al. (2015) used sounds congruent with the items to be remembered (e.g., an image of a cat presented with a “meow” sound), and the present study used sounds that were typical of an urban environment. Further work is necessary to determine whether arbitrary TMR cues are sufficient to demonstrate similar benefits to visuospatial memories. Finally, as mentioned previously, our aim was to demonstrate the feasibility of delivering CL-TMR during sleep and that improvements on a real-world task can result even after a relatively short nap. This study does not answer the question of whether the performance benefits observed after CL-TMR are greater than those seen after open-loop TMR, a method with cues delivered at random in relation to the slow oscillation phase. This study also did not examine cues timed to the up-to-down states; however, it is expected that those cues would be processed mainly during the down-state, resulting in a more limited performance benefit. In line with this assumption, Göldi et al. (unpublished) have shown that the benefit of TMR only reached statistical significance when the cues were delivered during the up-state in comparison to the down-state.

We have reported the first implementation of a CL-TMR approach to enhance navigation learning. We showed that our approach was able to induce DUPT-locked spindle activity and improve navigation efficiency in a large-scale VR-based task. These effects were obtained without any negative impact on sleep architecture. Overall, these findings suggest that CL-TMR is a feasible and effective technique to optimize the sleeping brain for memory processing. These results hold promise for the application of sleep-based interventions to drive improvement in real-world tasks.

## Author Contributions

SS, PC, MA, MW, and SM designed the experiment. RS, DA, NC, and LH collected the data. RE, MA, and SS developed the algorithms. SS and NC performed the statistical analyses. RS, NC, and SS wrote the manuscript. All authors contributed to manuscript revision and approved the submitted version.

## Conflict of Interest Statement

Teledyne Scientific & Imaging has filed a patent application (no. 62/403318) based on parts of the described research. RS, PC, RE, MA, and SS were employed by Teledyne Scientific & Imaging. DA and MW were employed by Rio Grande Neurosciences. The other authors declare that the research was conducted in the absence of any commercial or financial relationships that could be construed as a potential conflict of interest.

## Footnotes

1 http://openvibe.inria.fr/

2 http://biosig.sourceforge.net/index.html

3 https://sccn.ucsd.edu/eeglab/index.php

## References

[B1] Andrade K. C. Spoormaker V. I. Dresler M. Wehrle R. Holsboer F. Sämann P. G. (2011). Sleep spindles and hippocampal functional connectivity in human NREM sleep. *J. Neurosci.* 31 10331–10339. 10.1523/JNEUROSCI.5660-10.2011 21753010 PMC6623055

[B2] Batterink L. J. Creery J. D. Paller K. A. (2016). Phase of spontaneous slow oscillations during sleep influences memory-related processing of auditory cues. *J. Neurosci.* 36 1401–1409. 10.1523/JNEUROSCI.3175-15.2016 26818525 PMC4728733

[B3] Batterink L. J. Paller K. A. (2017). Sleep-based memory processing facilitates grammatical generalization: evidence from targeted memory reactivation. *Brain Lang.* 167 83–93. 10.1016/j.bandl.2015.09.003 26443322 PMC4819015

[B4] Benjamini Y. Hochberg Y. (2000). On the adaptive control of the false discovery rate in multiple testing with independent statistics. *J. Educ. Behav. Stat.* 25 60–83. 10.3102/10769986025001060 14584715

[B5] Born J. Rasch B. Gais S. (2006). Sleep to remember. *Neuroscientist* 12 410–424. 10.1177/1073858406292647 16957003

[B6] Buzsaki G. (1998). Memory consolidation during sleep: a neurophysiological perspective. *J. Sleep Res.* 7 S17–S23. 10.1046/j.1365-2869.7.s1.3.x 9682189

[B7] Clemens Z. Fabo D. Halasz P. (2005). Overnight verbal memory retention correlates with the number of sleep spindles. *Neuroscience* 132 529–535. 10.1016/j.neuroscience.2005.01.011 15802203

[B8] Creery J. D. Oudiette D. Antony J. W. Paller K. A. (2015). Targeted memory reactivation during sleep depends on prior learning. *Sleep* 38 755–763. 10.5665/sleep.4670 25515103 PMC4402655

[B9] Crunelli V. Hughes S. W. (2010). The slow (<1 Hz) rhythm of non-REM sleep: a dialogue between three cardinal oscillators. *Nat. Neurosci.* 13 9–17. 10.1038/nn.2445 19966841 PMC2980822

[B10] Delorme A. Makeig S. (2004). EEGLAB: an open source toolbox for analysis of single-trial EEG dynamics including independent component analysis. *J. Neurosci. Methods* 134 9–21. 10.1016/j.jneumeth.2003.10.009 15102499

[B11] Diekelmann S. Born J. (2010). The memory function of sleep. *Nat. Rev. Neurosci.* 11 114–126. 10.1038/nrn2762 20046194

[B12] Diekelmann S. Büchel C. Born J. Rasch B. (2011). Labile or stable: opposing consequences for memory when reactivated during waking and sleep. *Nat. Neurosci.* 14 381–386. 10.1038/nn.2744 21258327

[B13] Doeller C. F. King J. A. Burgess N. (2008). Parallel striatal and hippocampal systems for landmarks and boundaries in spatial memory. *Proc. Natl. Acad. Sci. U.S.A.* 105 5915–5920. 10.1073/pnas.0801489105 18408152 PMC2311337

[B14] Eschenko O. Mölle M. Born J. Sara S. J. (2006). Elevated sleep spindle density after learning or after retrieval in rats. *J. Neurosci.* 26 12914–12920. 10.1523/JNEUROSCI.3175-06.2006 17167082 PMC6674950

[B15] Gais S. Mölle M. Helms K. Born J. (2002). Learning-dependent increases in sleep spindle density. *J. Neurosci.* 22 6830–6834. 12151563 10.1523/JNEUROSCI.22-15-06830.2002 PMC6758170

[B16] Hartley T. Maguire E. A. Spiers H. J. Burgess N. (2003). The well-worn route and the path less traveled: distinct neural bases of route following and wayfinding in humans. *Neuron* 37 877–888. 10.1016/S0896-6273(03)00095-3 12628177

[B17] He J. Sun H.-Q. Li S.-H. Zhang W.-H. Shi J. Ai S.-Z. (2015). Effect of conditioned stimulus exposure during slow wave sleep on fear memory extinction in humans. *Sleep* 38 423–431. 10.5665/sleep.4502 25348121 PMC4335533

[B18] Iber C. Ancoli-Israel S. Chesson A. Quan S. (2007). *The AASM Manual for the Scoring of Sleep and Associated Events: Rules, Terminology, and Technical Specifications.* Westchester, IL: American Academy of Sleep Medicine.

[B19] Jarosz A. F. Wiley J. (2014). What are the odds? A practical guide to computing and reporting Bayes factors. *J. Probl. Solving* 7:2 10.7771/1932-6246.1167

[B20] John O. P. Donahue E. M. Kentle R. L. (1991). *The Big Five Inventory—Versions 4a and 54.* Berkeley, CA: University of California.

[B21] Lemieux M. Chen J.-Y. Lonjers P. Bazhenov M. Timofeev I. (2014). The impact of cortical deafferentation on the neocortical slow oscillation. *J. Neurosci.* 34 5689–5703. 10.1523/JNEUROSCI.1156-13.2014 24741059 PMC3988418

[B22] Leminen M. M. Virkkala J. Saure E. Paajanen T. Zee P. C. Santostasi G. (2017). Enhanced memory consolidation via automatic sound stimulation during non-REM sleep. *Sleep* 40 1–10. 10.1093/sleep/zsx003 28364428 PMC5806588

[B23] Mander B. A. Santhanam S. Saletin J. M. Walker M. P. (2011). Wake deterioration and sleep restoration of human learning. *Curr. Biol.* 21 R183–R184. 10.1016/j.cub.2011.01.019 21377092 PMC3093247

[B24] Marshall L. Born J. (2007). The contribution of sleep to hippocampus-dependent memory consolidation. *Trends Cogn. Sci.* 11 442–450. 10.1016/j.tics.2007.09.001 17905642

[B25] Ngo H.-V. V. Martinetz T. Born J. Mölle M. (2013). Auditory closed-loop stimulation of the sleep slow oscillation enhances memory. *Neuron* 78 545–553. 10.1016/j.neuron.2013.03.006 23583623

[B26] Ngo H.-V. V. Miedema A. Faude I. Martinetz T. Mölle M. Born J. (2015). Driving sleep slow oscillations by auditory closed-loop stimulation—a self-limiting process. *J. Neurosci.* 35 6630–6638. 10.1523/JNEUROSCI.3133-14.2015 25926443 PMC4412888

[B27] Niknazar M. Krishnan G. P. Bazhenov M. Mednick S. C. (2015). Coupling of thalamocortical sleep oscillations are important for memory consolidation in humans. *PLOS ONE* 10:e0144720. 10.1371/journal.pone.0144720 26671283 PMC4699460

[B28] Oudiette D. Paller K. A. (2013). Upgrading the sleeping brain with targeted memory reactivation. *Trends Cogn. Sci.* 17 142–149. 10.1016/j.tics.2013.01.006 23433937

[B29] Rasch B. Born J. (2013). About sleep’s role in memory. *Physiol. Rev.* 93 681–766. 10.1152/physrev.00032.2012 23589831 PMC3768102

[B30] Rasch B. Büchel C. Gais S. Born J. (2007). Odor cues during slow-wave sleep prompt declarative memory consolidation. *Science* 315 1426–1429. 10.1126/science.1138581 17347444

[B31] Rihm J. S. Diekelmann S. Born J. Rasch B. (2014). Reactivating memories during sleep by odors: odor specificity and associated changes in sleep oscillations. *J. Cogn. Neurosci.* 26 1806–1818. 10.1162/jocn_a_00579 24456392

[B32] Rudoy J. D. Voss J. L. Westerberg C. E. Paller K. A. (2009). Strengthening individual memories by reactivating them during sleep. *Science* 326 1079–1079. 10.1126/science.1179013 19965421 PMC2990343

[B33] Schabus M. Dang-Vu T. T. Albouy G. Balteau E. Boly M. Carrier J. (2007). Hemodynamic cerebral correlates of sleep spindles during human non-rapid eye movement sleep. *Proc. Natl. Acad. Sci. U.S.A.* 104 13164–13169. 10.1073/pnas.0703084104 17670944 PMC1941810

[B34] Schabus M. Gruber G. Parapatics S. Sauter C. Klosch G. Anderer P. (2004). Sleep spindles and their significance for declarative memory consolidation. *Sleep* 27 1479–1485. 10.1093/sleep/27.7.1479 15683137

[B35] Schmidt C. Peigneux P. Muto V. Schenkel M. Knoblauch V. Münch M. (2006). Encoding difficulty promotes postlearning changes in sleep spindle activity during napping. *J. Neurosci.* 26 8976–8982. 10.1523/JNEUROSCI.2464-06.2006 16943553 PMC6675334

[B36] Schreiner T. Rasch B. (2014). Boosting vocabulary learning by verbal cueing during sleep. *Cereb. Cortex* 25 4169–4179. 10.1093/cercor/bhu139 24962994

[B37] Siapas A. G. Wilson M. A. (1998). Coordinated interactions between hippocampal ripples and cortical spindles during slow-wave sleep. *Neuron* 21 1123–1128. 10.1016/S0896-6273(00)80629-7 9856467

[B38] Spielberger C. D. Gorsuch R. L. Lushene R. Vagg P. R. Jacobs G. A. (1983). *Manual for the State-Trait Anxiety Inventory.* Palo Alto, CA: Consulting Psychologists Press.

[B39] Staresina B. P. Bergmann T. O. Bonnefond M. Van Der Meij R. Jensen O. Deuker L. (2015). Hierarchical nesting of slow oscillations, spindles and ripples in the human hippocampus during sleep. *Nat. Neurosci.* 18 1679–1686. 10.1038/nn.4119 26389842 PMC4625581

[B40] Tamminen J. Ralph M. A. L. Lewis P. A. (2013). The role of sleep spindles and slow-wave activity in integrating new information in semantic memory. *J. Neurosci.* 33 15376–15381. 10.1523/JNEUROSCI.5093-12.2013 24068804 PMC3782619

[B41] Timofeev I. Bazhenov M. (2005). “Mechanisms and biological role of thalamocortical oscillations,” in *Trends in Chronobiology Research* ed. Columbus F. H. (Hauppauge, NY: Nova Science Publishers) 1–47.

[B42] van Dongen E. V. Takashima A. Barth M. Zapp J. Schad L. R. Paller K. A. (2012). Memory stabilization with targeted reactivation during human slow-wave sleep. *Proc. Natl. Acad. Sci. U.S.A.* 109 10575–10580. 10.1073/pnas.1201072109 22691500 PMC3387124

[B43] Wagenmakers E.-J. (2007). A practical solution to the pervasive problems of p values. *Psychon. Bull. Rev.* 14 779–804. 10.3758/BF03194105 18087943

[B44] Walker M. P. (2009). The role of sleep in cognition and emotion. *Ann. N. Y. Acad. Sci.* 1156 168–197. 10.1111/j.1749-6632.2009.04416.x 19338508

[B45] Wilhelm I. Kurth S. Ringli M. Mouthon A.-L. Buchmann A. Geiger A. (2014). Sleep slow-wave activity reveals developmental changes in experience-dependent plasticity. *J. Neurosci.* 34 12568–12575. 10.1523/JNEUROSCI.0962-14.2014 25209294 PMC6615503

[B46] Wilson M. A. McNaughton B. L. (1994). Reactivation of hippocampal ensemble memories during sleep. *Science* 265 676–679. 10.1126/science.8036517 8036517

[B47] Yordanova J. Kirov R. Verleger R. Kolev V. (2017). Dynamic coupling between slow waves and sleep spindles during slow wave sleep in humans is modulated by functional pre-sleep activation. *Sci. Rep.* 7:14496. 10.1038/s41598-017-15195-x 29101344 PMC5670140

[B48] Zeitlhofer J. Gruber G. Anderer P. Asenbaum S. Schimicek P. Saletu B. (1997). Topographic distribution of sleep spindles in young healthy subjects. *J. Sleep Res.* 6 149–155. 10.1046/j.1365-2869.1997.00046.x 9358392

